# Improvement of Emergency Management Mechanism of Public Health Crisis in Rural China: A Review Article

**Published:** 2018-02

**Authors:** Jiaxiang HU, Chao CHEN, Tingting KUAI

**Affiliations:** 1. College of Economics and Management, Nanjing Agricultural University, Nanjing, China; 2. China Center for Food Security Studies, Nanjing Agricultural University, Nanjing, China

**Keywords:** Rural area, Public health crisis, Emergency management, Organization mechanism, Operation mechanism

## Abstract

**Background:**

With the rapid development of social economy in China, various public health emergencies frequently occur. Such emergencies cause a serious threat to human health and public safety, especially in rural China. Owing to flaws in emergency management mechanism and policy, the government is not capable to effectively deal with public health emergencies. Therefore, this study aimed to discuss the path to improve the emergency management mechanism for public health emergency in rural China.

**Methods:**

This study was conducted in 2017 to detect the emergency management mechanism of public health crisis (EMMPHC) in Rural China. Data were collected using the following keywords: Rural China, public health emergency, emergency management mechanism, organization mechanism, operation mechanism in the databases of PubMed, Scopus, Web of Science, and CNKI.

**Results:**

EMMPHC in rural China can be enhanced from the following three aspects. First, a permanent institution for rural emergency management with public health management function is established. Second, the entire process of emergency management mechanism, including the stages of pre-disaster, disaster, and post-disaster, is improved. Finally, investment in rural public health is increased, and an adequate reserve system for emergency resources is formed.

**Conclusion:**

The new path of EMMPHC in rural China can effectively help the local government accomplish the dispatch capability in public health emergency, and it has important research significance for the protection of public health and social stability of residents in rural China.

## Introduction

With the rapid development of China’s economy, various public health emergencies from traditional and nontraditional fields have also become increasingly prominent. An example of such emergency is the outbreak of avian influenza H7N9 infection in the spring of 2017, which spread to Beijing, Fujian, Yunnan, Hunan, Hubei, Zhejiang, and other provinces within a few months (1). This outbreak is the most serious since the first outbreak in China in 2013, thereby causing the largest number of infections in five years (1). According to an uncompleted statistic, 32 kinds of new infectious diseases have been discovered in the last 20 years, approximately half of which have appeared in China (2). In rural areas of China, especially in the poor areas of Western China, the prevalence of infectious and endemic diseases incidence is high (2). Approximately 60% of the Chinese population comprises farmers living in the vast countryside. Rural areas have become vulnerable communities to all kinds of disasters (3), where the health and public safety of residents and the rural society are also most vulnerable to infringement. Therefore, strengthening the effective prevention and timely dispatch of public health emergency in rural areas is vital. For this reason, the emergency management mechanism in rural areas has gradually become the focus of the government and society.

Public health emergency regulations in China define public health emergencies as “unexplained diseases, major food or drinking water and occupation poisoning, and other serious public health events which suddenly occur, thereby causing major infectious diseases or even serious damage to public health groups” (4). The emergency management mechanism is the method and measure of the government in handling an emergency (5), and such mechanism includes structure and function systems. The structural system deals with emergencies, including four systems of processing mechanism: decision-making, information, implementation, and security. Meanwhile, the function system involves different stages of response measures to the periodic fluctuation of public health emergencies and includes the following five aspects: prevention, reaction, diffusion, recovery, and summarization. The functional system is also an important external dominant feature of the structural system (6).

Rural public crisis management is a complex system of engineering and involves a multitude of things. Hence, if the government does not consider the scientific management method in public crisis management, then exploring the perfect mechanism for crisis management in a brief period of time is very difficult (7). However, owing to different geographical environment and cultural soil of the countryside and the city, many scholars believe that public crisis management mechanisms in rural areas cannot simply copy those of the city (7).

This study mainly focuses on means of improving the emergency management mechanism of public health emergency (EMMPHE) in rural China. Moreover, this study will provide a reference for improving emergency preparedness and dispatch of public health emergency in rural China, thereby expanding to new fields of emergency management.

## Methods

The researchers independently conducted a literature search of PubMed, Scopus, Web of Science, CNKI databases; all included papers were published in English or Chinese before Sep 2017. Citations meeting inclusion criteria were full screened, and available data were abstracted independently. Public health and epidemic prevention system should include six subsystems of doctors in epidemic prevention, epidemic monitoring, disease analysis, emergency response, compensation, and material support (8). The qualitative analysis framework of the capability evaluation of crisis management for the local government was constructed in four stages: preparation in the earlier stage, mitigation in the medium term, and response and recovery in the latter stages (9). Effective bioterrorism preparedness in rural communities was a significant issue (3), and the emergency preparedness of all workgroups within rural public health departments was examined (10). In addition, the preparedness of rural health care professionals was important in case of a bioterrorist attack or other public health emergencies (11). In emergency response, taking timely preventive measures against avian influenza remarkably affected control and reduction of damage caused by the animal epidemic for the government (12). A participatory emergency preparedness training program was developed, which was feasible and effective in improving the performance of rural public health personnel (13). In addition, the evaluation and monitoring of emergency management training programs and exercises were conducted (14). To establish public health policies, emergency medical service (EMS) was explored, and each EMS team should be equipped with adequate emergency care facilities and well-trained personnel (15). For boosting emergency response willingness among other cadres of health providers, the potential applicability of training curriculum was necessary (16). In the recovery phase, the Public Health Assessment for Emergency Response (PHASER) Toolkit was used to rapidly assess health needs of households in urban and rural areas (17).

## Results and Discussion

The literature review shows that existing studies focus on single crisis management strategies of local governments. Such strategies can help local governments improve the emergency management mechanism of public health emergencies. However, the emergency management of rural public health emergencies involves various strategies with strong practical applications. Therefore, based on the analysis of operating practices of foreign countries, the scientific management method can be used to propose the path for the systematic and comprehensive improvement of EMMPHE. However, no research in this area has been conducted yet. Based on the entire process of crisis management, including disaster prevention, response, and recovery, the path of improving EMMPHE in rural areas is systematically and integrally proposed, which is different from previous ones.

### Problem analysis on the emergency management mechanism of rural China

The management mechanism in rural areas is problematic in three aspects: organization, operation mechanism, and guarantee system.

### Lack of permanent comprehensive emergency response agency

The emergency management system in rural areas mainly refers to the division of power and responsibility between the county and higher governments, between the county and township governments, and between different organizations within the county and its township government. In accordance with the current countryside situation, the county government has established an emergency office as a comprehensive coordination organization. The duties of this office include “providing assistance to the government during public emergencies; coordination and guidance for the prevention and early warning of emergencies; supervision of emergency handling, investigation, and assessment; and security work in public emergencies (18).” Once unexpected public health incidents occur in rural areas, the county health department will be responsible for the establishment of temporary emergency command. Hence, the function of the emergency office and temporary emergency command in handling the emergency overlaps to a certain extent, thus easily leading to unclear responsibilities. Coordination between the two institutions is also problematic, thus impeding effective management of public health emergencies (2).

### Imperfections of the rural emergency management mechanism

#### 1) Inadequate prevention and preparation prior to the disaster

Prevention and preparation prior to the disaster involve a wide range of strategies. One of the most effective and practical strategies is preparing various emergency plans (19). Approximately 98.7% of county governments completed the formulation of emergency plans (20). However, crisis awareness and education in rural areas are deficient due to shortage of manpower, organization, technology, equipment, as well as resources such as social capital; ineffective training and formulation of emergency plans also contribute to this deficiency. Table 1 reflects the emergency training situation of rural residents of a province in China prior to disaster (21). The table shows that most rural residents rarely participate in emergency training, drills, and publicity as well as educational activities. In addition, farmers are often excluded from participating in prevention and preparedness work prior to the disaster in consideration of departmental interests. Consequently, risk in rural areas will significantly increase, and the capability of rural society to prevent and resist various crisis events will be reduced (22).

**Table 1:** Emergency training of rural residents of a province in China prior to disaster

***Variable***	***Value***	***Valid Percentage***	***Mean±SD***
Frequency of participation in first aid training	Never	76.3	11.2±2.1
	One or two times	13.7	
	Three times or more	0	
Frequency of participation in emergency drills	Never	80.2	11.0±2.0
	One or two times	19.8	
	Three times or more	0	
Frequency of participation in propaganda for emergency knowledge	Never	73.9	11.3±2.2
	One or two times	26.1	
	Three times or more	0	
Frequency of participation in emergency rescue teams	Never	78.9	11.0±2.0
	One or two times	21.1	
	Three times or more	0	
Frequency of participation in disaster risk inspection	Never	72.7	11.4±2.2
	One or two times	23.3	
	Three times or more	0	

#### 2) Weakness in coping with disaster

Coping mechanisms in public health emergencies include early warning in the initial stage and emergency response in the stage of large-scale outbreak. If people detect information on the epidemic at the initial stage, the large-scale outbreak of the epidemic can be avoided due to timely alert and rapid intervention measures (23, 24). Owing to the under development of rural areas, problems in the early warning of public emergencies exist, such as lack of awareness regarding early warning, low degree of attention (25), a single method of crises monitoring, formalization of information report, and difficulty in transmission.

Emergency response is key to the entire crisis management and is the most centralized and urgent stage for all kinds of management resources. Owing to the limited quality and capability of three-level organizations and leaders in counties and villages, rural grass-root organizations and autonomous forces “hollowing” in the face of disasters has become the norm (26). In addition, a considerable amount of information related to the vital interests of farmers which ought to be disclosed are often classified as confidential by the county government and are closed to the public. All these problems have seriously restricted the improvement of rural emergency response capabilities.

#### 3) Imperfect mechanism of recovery after the disaster

The current recovery mechanism after the disaster only includes material compensation for victims of such disaster, thereby ignoring their psychological recovery and the investigation responsibility for the incident. Even material compensation only has a single standard of compensation, which seriously ignores the specific damage situation of victims. For example, in the incident of Sanlu milk contamination, children received compensation in accordance with three cases, namely death and severe and general injuries, as determined by the Chinese Ministry of Health. In this condition, some children that presented mild symptoms could only receive 2000 Yuan even when actual medical expenses exceeded more than such amount (27). If the psychological trauma of the victim and his or her family is not promptly addressed, then negative effects from such trauma will permanently afflict these victims. Incidence of depression is increasing after the incidents (28). Moreover, considering limited performance evaluation index systems in rural areas, the county government is not liable for any responsibility on the matter. Hence, implementing duties through the grass-roots unit of the Chinese government system is difficult.

### Low investment in rural public health and weak capacity of emergency support

The operation of rural public health service system has been mainly borne by local government. However, investment in rural public health is deficient due to the financial stress of the county and township governments. Table 2 reflects the comparison of per capita health expenditure between urban and rural residents in 2012, 2013, and 2014. A big gap evidently exists between urban and rural public health resources allocation in China. Thus, rural areas have long been plagued by the shortage of medical and health resources. Rural areas also still lack well-trained public health workers, which mainly include practitioners, assistant doctors, registered nurses, and other staff directly engaged in medical and health services. Table 3 reflects the number of technical health personnel per 1000 population in urban and rural areas in 2013, 2014, and 2015. The numbers of technical health personnel per 1000 population in the city are found to have reached 2.6 times of that in rural areas, and a big gap exists between urban and rural resources. In addition, statistics of developed countries indicate that more than 10 medical staffs per 1000 population in rural areas are represented, and a big gap also exists between China and the international community.

**Table 2:** Comparison of per capita health expenditure between urban and rural residents in 2012, 2013, and 2014

***Year***	***Per capita city health expenditure (USD)***	***Per capita country health expenditure (USD)***	***City/Country (Times)***	***Country/Nation (Times)***	***City/Village (Times)***
2012	476.08	169.02	1.44	0.51	2.82
2013	514.17	202.61	1.39	0.55	2.54
2014	581.42	230.75	1.38	0.55	2.52

Sources: 2016 China Statistical Yearbook

**Table 3:** Comparison of technical health personnel per 1000 population in urban and rural areas in 2013, 2014, and 2015

***Year***	***Number of health technical personnel per 1000 population***			***Number of licensed physicians per 1000 population***			***Number of registered nurses per 1000 population***		
	**City**	**Village**	**City/Village**	**City**	**Village**	**City/Village**	**City**	**Village**	**City/Village**
2013	9.18	3.64	2.52	3.39	1.48	2.29	4.00	1.22	3.28
2014	9.10	3.77	2.41	3.54	1.51	2.34	4.30	1.31	3.28
2015	10.0	3.90	2.62	3.70	1.60	2.31	4.60	1.40	3.29

Sources: 2016 China Statistical Yearbook

Meanwhile, related emergency supplies, technology, and reserves are seriously inadequate, thereby restricting emergency guarantee capability. For example, 31 provinces (autonomous regions and municipalities) generally had formed a provincial reserve system, and 75.3% of prefecture-level cities had established a municipal pool. However, only 56.5% of counties (cities) had reserve storage, and storage of rural relief materials in counties and areas below county-level were basically empty (20). The lack of not only emergency materials and reserve facilities but also trained public health workers with regularity will seriously affect the handling of emergencies and the development of public health.

### Experience and enlightenment from foreign countries

The capability of the United States to manage public health emergencies is considered the best in the world, as indicated in the Political & Economic Risk Consultancy 2012 report (29). Japan, who frequently encounters disasters, has a relatively mature crisis management system. India and China are both developing countries with large populations and their experiences are more closely related.

### United States

EMMPHE in the United States comprises three levels, which include federal, state, and county (city), and is connected to other systems (such as energy, environment, and other systems) (30). The main body of the public health emergency in the United States is federal disease control and prevention system, state hospital emergency preparedness system, and local urban medical coping system. Among these systems, the United States Centers for Disease Control and Prevention(CDC) is not only the core management organization and coordination center of public health emergencies but also is the specific decision-making organization, which is affiliated with the US Department of Health (31).

For general emergencies, the Department of Homeland Security has a major leadership role in coordinating federal agencies in dealing with public emergencies. The Federal Emergency Management Agency has become a coordinating body for all emergency operations, and all sources of information are imported into this organization. No matter what kind of decision-making model, the CDC-based three-level response system is responsible for the specific implementation of emergency work, and the horizontal and vertical cooperation among agencies is emphasized in the implementation process (32).

Since the 1970s, the United States has established and improved the national crisis response system based on the National Security Law, National emergencies Act, and the other core legal systems. In addition, the United States has a set of resources guarantee system in response to public health emergency, including National Notifiable Diseases Surveillance System, Laboratory Response Network (LRN), and the National Urban Search and Rescue Response System et.al.

### Japan

The organization system of Japan for public health emergency comprises the Ministry of Health, Sub-Bureaus of Health, National University of Medicine School and its Affiliated Hospital, National Hospital, National Sanatorium, and National Research Institute. The local government of emergency management system is composed of the municipal health and health bureau, care center, health laboratory, county hospital, health center of village and town (33).

The emergency operation mechanism in Japan is based on the legal system of emergency health emergency and is a coordination mechanism of multisystem, multilevel, and multidisciplinary through resource security, information management, and health education systems. Through the vertical industry system and regional management, national and local emergency systems have formed a national emergency management network for public health emergency.

Japan has established a sound legal system of emergency management and promulgated the Disaster Countermeasures Basic Act, which can effectively improve the capability and level of overall emergency management of Japan (34). The emergency resources guarantee system of Japan includes the following three aspects: personnel, capital, and materials. The emergency personnel team comprises specially trained full-time staff and part-time volunteers, and the proportion of national burden in emergency treatment is clearly defined by legislation. For material support, emergency materials reserve and periodic rotation system have been established, and Japanese families reserve emergency supplies and self-help appliances for disaster prevention.

### India

The organization of emergency management in India is divided into four levels, namely federal, state, county, and district and the governments at these four levels have established unified disaster management institutions. The federal is the core, and the central government is mainly responsible for the coordination of resources and other support work. The government departments at all levels basically set up command centers, which will function once the crisis report is launched. In order to ensure the timely start of disaster relief operations, the government of India established and periodically updates the Emergency Action Plan (33), which defines the specific action plan taken by the central ministries and bureaus after the disaster.

Due to different national conditions, obtaining the experience according to the actual situation of the country is necessary. The United States has a perfect public emergency organization system and coordination mechanism and has formed a comprehensive, three-dimensional, and coordinated disaster management system with the perfect emergency legal system as the guarantee. Owing to its powerful emergency command center, the public crisis emergency response efficiency of Japan is very high. Meanwhile, the management network system comprising local health care centers, which are equipped with a variety of strategic mutual aids and self-help systems, as well as the development of education, also provide a good guarantee for the system to play an effective role. India and China have numerous similarities, such as large population, vast territory, various disasters and frequent occurrence, thereby making it extremely easy to cause outbreak of large-scale epidemic due to public crisis. Therefore, the dynamic emergency treatment model of India for public crisis events has a high reference value for China, especially in rural areas.

### Result analysis and discussion of the means to improve the management mechanism of public health emergencies in rural areas

This study analyzes EMMPHE in rural China from the following three aspects: organizational mechanism, process management, and guarantee system.

### Establishing an efficient emergency organization system

Sudden public health incidents in rural areas have a wide range of hazards that are difficult to effectively control and handle when solely relying on departments of the county government. In this regard, the emergency management system of the United States provides a reference for China. For example, emergency management can be normalized to coordinate the government and its functional departments at all levels. Thus, the function of public health management in permanent institutions of disaster management can be strengthened. Additionally, a Committee of Experts is appointed as a special working body, and its responsibilities and division of labor are clearly defined. Only through this can various forces in society be used to their full extent in an effective and coordinative manner to cope with disasters and avoid mutual prevarication between functional departments and governments at various levels during disasters.

### Improving the entire course management mechanism 1) Strengthening awareness of disaster prevention and preparedness before disaster

The mechanism of prevention and preparedness prior to disasters can be perfected from the following two aspects. On one hand, subjective participation consciousness of farmers shall be emphasized, and emergency management in rural areas cannot entirely rely on the government. As the key members in rural areas, farmers ought to have the consciousness of subject participation, mobilize and integrate forces of village organizations, and carry out prevention and rescue work. On the other hand, various forms of targeted activities for publicity and education ought to be carried out. In the process of crisis education, imparting knowledge and skills to the villagers according to their cultural characteristics and actual needs is necessary because they are the main object of rural crisis education.

### 2) Enhancing the education of early warning and response during the disaster

Strengthening publicity and improving and enhancing the emergency consciousness of farmers are vital to a successful emergency management in the early warning stage of social emergencies. In addition, psychological preparation for emergencies shall be well executed through the education of farmers, and early warning management shall be strengthened through scientific prevention (10). Allowing media and the public to receive the latest news during an emergency is vital to strengthen the publicity and education of rural residents on public health emergencies, enhance knowledge of responses to rural public health emergencies (13), and improve the willingness of farmers to report on various epidemic events. Moreover, through extensive propaganda, the government shall help farmers understand their duty in public health emergency and allow them to actively participate in various emergency drills or exercises. Only by combining the decision-making of government with the masses can the capability of rural areas to cope with public health emergencies be effectively enhanced.

### 3) Emphasizing the restoration of physical and mental health of farmers and the supervision and evaluation of events after the disaster

After the public health emergency, the local government shall appoint relevant personnel to formulate the legal compensation system. Apart from the legal compensation system, the supervision and evaluation mechanism for the disaster shall also be established for timely comprehension of the implementation of control measures for public health emergencies before, during, and after the disaster. Such comprehension can help identify effective prevention and treatment measures and act as a basis for similar incidents. Although macro evaluation index of public health emergencies and micro evaluation index of emergency capabilities of CDC exist (35), emergency capability evaluation index system of grass-roots government in rural areas is absent. Therefore, comprehensive evaluation tools and scientific evaluation of public health emergency response capacities shall be developed.

### Increasing the investment in public health and establishing an adequate reserve system

Emergency guarantee mechanism is the foundation of establishing and perfecting the public health emergency management mechanism, which can be enhanced from investment in public health and the establishment of an emergency resource reserve system. Governments at all levels need to further improve the implementation of the fiscal subsidy policy of the public health agency. This improvement may be achieved by increasing the investment in public health and taking the main responsibility for public health to promote the realization of social justice as well as coordinated and balanced development among regions (36). Additionally, county and township governments can determine specific allocation of funds, thereby allowing service institutions to obtain reasonable compensation for service cost and reduce the burden of service objects (37). As for rural emergency guarantee, the reserve system of emergency resources can be established and improved. On the one hand, layout of the national material reserve system can be further improved, and construction of national material reserve systems can be extended from eastern and middle parts of China to western parts as well as from county-level and below. On the other hand, variety in reserve supplies can also be increased. Such increase can not only satisfy the needs of emergency rescue, disaster relief, and resettlement of victims but also allow preparation for conditions of disaster recovery and reconstruction planning.

## Conclusion

Improving the capability to handle and prevent public health emergencies is a problem that must be considered by the local government in China. In this study, literature analysis was used to perfect methods for the enhancement of the emergency management mechanism of rural China. These new methods can improve the capability of local government to handle and prevent public health emergencies from the following aspects. First, the permanent standing body for emergency management in rural areas could provide a powerful organization for disaster response. Second, sound management mechanism in the stages of pre-disaster, disaster, and post-disaster could provide an effective method for dealing with the disaster. Finally, continued investment in public health and adequate reserves of emergency supplies could provide a solid guarantee for residents of rural area. Perfecting the emergency management mechanism of rural public health emergency is of considerable significance to ensure public health and safety of farmers as well as the stability of rural society.

## Ethical considerations

Ethical issues (Including plagiarism, informed consent, misconduct, data fabrication and/or falsification, double publication and/or submission, redundancy, etc.) have been completely observed by the authors.
